# Construct Validity and Psychometric Properties of the Hebrew Version of the City Birth Trauma Scale

**DOI:** 10.3389/fpsyg.2018.01726

**Published:** 2018-09-18

**Authors:** Jonathan E. Handelzalts, Ilana S. Hairston, Adi Matatyahu

**Affiliations:** ^1^School of Behavioral Science, The Academic College of Tel Aviv-Yaffo, Yaffo, Israel; ^2^Department of Psychology, Tel-Hai Academic College, Qiryat Shemona, Israel; ^3^Psychiatry Department, University of Michigan, Ann Arbor, MI, United States

**Keywords:** peripartum disorders, childbirth, Hebrew, PTSD, postpartum

## Abstract

As many as third of the women perceive their childbirth as traumatic and although prevalence rates vary between studies, around 2–5% of women in community samples may develop childbirth-related postpartum post-traumatic stress disorder (PPTSD). The City Birth Trauma Scale (BiTS) was developed to address the need for a DSM-5-based instrument that assesses PPTSD. The BiTS is a self-report questionnaire, which covers all DSM-5 PTSD criteria, including the four symptom clusters – re-experiencing, avoidance, negative mood and cognitions and hyperarousal symptoms. The present study aimed to describe the psychometric properties and validate the Hebrew version of the BiTS. Five hundred and four mothers of 0- to 12-month-old infants were sampled using social media and the snowball method. Respondents completed an online survey consisting of a demographic questionnaire and the Hebrew versions of the BiTS, the impact of event scale-revised (IES-R), the Edinburgh postpartum depression scale (EPDS), and the Pittsburgh Sleep Quality Index (PSQI). The Hebrew BiTS demonstrated high internal consistency for the total scale (Cronbach α = 0.90) and good internal consistency (Cronbach’s α = 0.75–0.85) for the subscales. An exploratory factor (EFA) analysis yielded a two-factors solution, accounting for 45% of variance, with general symptoms loaded on Factor 1, and childbirth-related symptoms loaded on Factor 2, with both factors demonstrating high internal consistency (Cronbach’s α = 0.90, 0.85, respectively). High convergent validity for the symptom cluster subscales was demonstrated with the parallel IES-R subscales, EPDS and PSQI. A two-step cluster analysis indicated that dysphoric and hyperarousal symptoms best differentiated the severity of symptoms of respondents across measures. In sum, the Hebrew BiTS was psychometrically sound, indicating its utility for clinical and non-clinical research. The EFA and cluster analyses support the differentiation between symptoms of dysphoria and hyperarousal from trauma (i.e., childbirth) specific symptoms, suggesting that symptoms relating to specific aspects of the trauma differ qualitatively from general symptom in the phenomenology of PPTSD. Further research using clinical samples and comparing the BiTS to DSM-5 diagnosis using clinical interview is needed.

## Introduction

While having a child is usually a joyful event, childbirth itself may be a traumatic experience (Dekel et al., 2017). Indeed, a growing body of evidence indicates that as many as a third of women rate their childbirth as psychologically traumatic (Ayers and Pickering, 2001), with some developing postpartum post-traumatic stress disorder (PPTSD, Ayers et al., 2016; Dekel et al., 2017; Yildiz et al., 2017).

According to the Diagnostic and Statistical Manual of Mental Disorders 5 (DSM-5, American Psychiatric Association [APA], 2013), PTSD is defined as a disorder that develops in response to an event associated that elicited real or perceived threat of death or physical harm to the person or to others, with four clusters of symptoms characterizing the disorder: re-experiencing aspects of the event, persistent avoidance of reminders of the event, negative cognitions and mood, and hyperarousal. Although childbearing is no longer inherently life-threatening, and the emotions associated with it are not a priory negative, for some women childbirth generates an experience of threat of severe physical harm or death to themselves or their newborn, thereby fulfilling the first PTSD criterion (e.g., Grimes, 1994; Boorman et al., 2014; Polachek et al., 2016). Further, unlike other instances of PTSD, where patients may avoid trauma-associated cues thereby limiting their anxiety, the baby is an unavoidable reminder of the traumatic birth, resulting in some women reporting feelings of guilt for blaming their child for their traumatic childbirth experience (Alder et al., 2006). Thus, intrusions (i.e., negative cognitions) and negative emotions, would be expected to be a central feature of PPTSD.

In general, PTSD is known to have comorbidity with depression and anxiety disorders (e.g., Grekin and O’Hara, 2014; Horesh et al., 2017), increasing the risk for postpartum psychiatric morbidity among women with PPTSD. Further, maternal PTSD may negatively impact maternal and spousal relationships (Ayers et al., 2006; Parfitt and Ayers, 2009; Hairston et al., 2018), and may even have long-term negative effects on infant behavior and development (Hairston et al., 2011); all of which underscore the importance of understanding and minimizing the risk of PPTSD.

The incidence of PPTSD ranges from 1.3 to 2.4% at 1–2 months postpartum, and up to 4.6% 3–12 months postpartum (Polachek et al., 2016; Dekel et al., 2017). However, it should be noted that assessment of PPTSD incidence is complicated by the fact that the literature often fails to differentiate childbirth related-PPTSD from postpartum post-trauma symptoms due to other antecedent trauma exposure (Dekel et al., 2017). Nevertheless, a recent systematic review of community samples from several countries confirmed that 4.6% of women had acute childbirth related-posttraumatic symptoms, while 1.8% developed chronic PPTSD, with an overall incidence of 2.4% (Dekel et al., 2017).

Despite its relatively high prevalence, childbirth related-PPTSD is still largely under-recognized in maternity services and, unlike postpartum depression, is not routinely screened for (Ayers et al., 2018). One barrier to screening for PPTSD is the dearth of validated questionnaires designed to evaluate the disorder. To date, two questionnaires have been developed specifically for PPTSD. The first, the Traumatic Event Scale (TES) by Wijma et al. (1997), based on DSM-IV criteria (DSM-IV, American Psychiatric Association [APA], 1994), which has been used in a substantial number of perinatal publications (e.g., Soet et al., 2003; Söderquist et al., 2009) yet lacks psychometric or predictive validation. The second, the Perinatal PTSD Questionnaire (PPQ, Quinnell and Hynan, 1999; Callahan et al., 2006), identifies some but not all DSM-5 PTSD symptom clusters, and is therefore not appropriate for diagnostic purposes. Thus, to date no questionnaire has been developed that assesses postpartum PTSD which is fully consistent with DSM-5 diagnostic criteria.

To address this gap, Ayers et al. (2018) developed the Birth Trauma Scale (BiTS). This scale is comprised of 31 items, 29 of which map onto DSM-5 diagnostic criteria, and additional questions for assessing DSM-IV criterion A2 and symptoms of emotional numbing. The scale was successful in identifying women with significant distress and impairment, and demonstrated good sensitivity, specificity and accuracy for PTSD diagnosis (Ayers et al., 2018). The aim of the current study was to assess psychometric properties of the Hebrew version of the City BiTS, in a community sample, and to assess its the construct validity of the its symptom cluster subscales. Specifically, construct validity of ‘re-experiencing,’ ‘avoidance’ and ‘hyperarousal’ items were tested against subscales of the Revised Impact of Events Scale (IES-R, Weiss and Marmar, 1997). Construct validity of negative mood and cognitions items were tested against the Edinburgh Postpartum Depression Scale (EPDS, Cox et al., 1987); and hyperarousal items were additionally tested against the Pittsburgh Sleep Quality Index (PSQI, Buysse et al., 1989).

## Materials and Methods

### Procedure and Participants

Ethical approval for this study was obtained from the Tel Aviv-Yafo Academic College IRB. The findings reported are based on the responses of a cross-sectional sample of 504 mothers of infants ages 0–12 months who completed the survey in full. Women were recruited using social media (e.g., Facebook, WhatsApp). Inclusion criteria were a singleton pregnancy within the previous 12 months. After providing consent, women completed the Hebrew versions of the following measures: A demographic questionnaire, The City Birth Trauma Scale (BiTS), The Impact of Event Scale - Revised (IES-R), The Edinburgh Postnatal Depression Scale (EPDS), and the Pittsburgh Sleep Quality Index (PSQI). Questionnaires and data output were generated using Qualtrics 2015 (Qualtrics^1^, Provo, UT, United States).

### Instruments

#### The Demographic Questionnaire

The demographic questionnaire contained questions regarding the participants’ age, ethnicity, education, and marital status. Respondents also answered questions regarding the pregnancy, such as medical complications during pregnancy, pregnancy at risk, and week of birth, time since birth, and a question regarding type of birth, i.e., vaginal, assisted vaginal, emergency cesarean or elective cesarean.

#### The Birth Trauma Scale (BiTS; Ayers et al., 2018)

The BiTS is a self-report questionnaire consisting of 31 items developed on the basis of DSM-5 (American Psychiatric Association [APA], 2013) criteria for PTSD, with additional symptoms that arose from interviews with women and experts in the field. Twenty-three of the items assess frequency of symptoms over the last week, scored on a Likert-type scale ranging from 0 (‘not at all’) to 3 (‘5 or more times’). These 23 items cover four symptom clusters of DSM-5: ‘re-experiencing’ symptoms (five items), ‘avoidance’ symptoms (two items), ‘negative mood and cognitions’ (seven items), and ‘hyperarousal’ symptoms (six items). Additionally, two items assessed criterion A in accordance with DSM-5 (American Psychiatric Association [APA], 2013) and another item assessed criterion A2 from DSM-IV (American Psychiatric Association [APA], 1994), namely believing that serious harm or death may occur, and experiencing intense negative emotions, scored as yes/no. Three items assessed degree of distress, disability and potential physical causes, scored as yes/no/maybe, and two items assessing onset (before/in the first 6 months/6 months after giving birth) and duration (less than 1 month, 1–3 months, more than 3 months) of symptoms. In the original study, the BiTS demonstrated high internal consistency (Cronbach’s α = 0.92), and Cronbach’s α = 0.90 for the Hebrew version in the current study. Translation and cultural adaptation procedures were in accordance with Brislin’s (1986) guidelines.

#### Impact of Event Scale-Revised (IES-R; Weiss and Marmar, 1997)

The IES-R is a 22-item measure designed in accordance with DSM-VI (American Psychiatric Association [APA], 1994) symptoms criteria for PTSD. Respondents were asked to rate each item on a scale of 0 (*not at all*) to 4 (*extremely*), according to severity of their symptoms of intrusion, hyperarousal, and avoidance, over the past 7 days. In the present study, the Hebrew version (Somer et al., 2005) was used, yielding Cronbach’s coefficients of α = 0.87 for Intrusion, α = 0.87 for Avoidance, and α = 0.85 for Hyperarousal.

#### Edinburgh Postnatal Depression Scale (EPDS; Cox et al., 1987)

The EPDS was developed as a screening tool for postpartum depression, consisting of 10 items rated on a 4-point scale, ranging from 0 to 3, with a maximum score of 30. Higher scores reflect greater risk for depression. A score over 10 indicates symptoms of depression and a score over 12 indicates significant depressive symptoms (Cox et al., 1987; Murray and Carothers, 1990). In the present study, the Hebrew version (Glasser and Barell, 1999) was used, obtaining Cronbach’s coefficient of α = 0.87.

#### Pittsburgh Sleep Quality Index (PSQI; Buysse et al., 1989)

The PSQI is a 19-item questionnaire developed to measure sleep quality and sleep disturbance. It assesses seven components of sleep (subjective sleep quality, sleep latency, sleep duration, habitual sleep efficiency, sleep disturbance, use of sleeping medications, and daytime dysfunction) in the prior 4 weeks. Each of the components has a score range of 0–3, and the global sleep quality score is obtained by summing the seven components. A total score >5 indicates insufficient sleep quality. The Hebrew version (Shochat et al., 2007) of the PSQI was used, obtaining Cronbach’s coefficient of α = 0.63, between components.

#### Statistical Analyses

Statistical analyses were done using SPSS V23. ANOVAs were used for bivariate or multivariate analyses where appropriate. An exploratory factor analysis (EFA) was conducted to determine the factor structure of the BiTS using only the items in the symptoms scale. Although the scale assumes the existence of four constructs – re-experiencing, avoidance, negative cognitions and mood, and hyperarousal – we opted for an EFA due to the lack of previous exploration of the measure. In accordance with Fabrigar et al. (1999) we employed maximum likelihood estimation and a ‘Direct Oblimin’ (oblique) rotation to allow factors to correlate. The scree plot of ordered eigenvalues of a correlation matrix was used to determine the appropriate number of factors extracted. A factor loading of >0.30 was used to select items for each factor, and Cronbach’s alpha was calculated for the internal consistency of the generated factors.

Construct validity was assessed by examining the relationship of the BiTS subscales and factors with other dependent measures. Two methods were employed; first zero-order correlations were used to determine similarity with other measures. Then a two-step cluster analysis (TSCA) was used to assess clusters, within the data set, based on the distribution of the BiTS subscales, the EPDS and sleep measures. The TCSA is an exploratory tool, that can handle a mix of categorical and continuous variables, and designed to reveal ‘natural’ clusters within a dataset that are not a priory hypothesized. Unlike other statistical techniques, cluster analysis does not identify a particular statistical model, it is simply allows the classification of homogeneous groups within complex data sets (Borgen and Barnett, 1987). For the analysis, we let the procedure automatically determine the number of clusters, using log-likelihood distance measure and the Schwarz’s Bayesian Criterion (BIC) as clustering criterion. To determine stability of clusters, the dataset was randomly split in two, and the procedure was re run on the two halves of the sample.

## Results

### Demographics

Of 594 entries, 504 women completed all questionnaires in full. Respondents ages ranged from 20 to 44, with infants ages ranging from 1 week to 13 months. For the majority (63%) this was their first child. The plurality had a normal pregnancy and vaginal birth without intervention, at 37 weeks or later (**Table 1**). Descriptive statistics of the self-report instruments (not including BiTS) are reported in **Table 1**. With respect to the IES-R, although it does not have recommended cutoffs, the total score was clearly below cutoff for a probable diagnosis of PTSD (Creamer et al., 2002). For the EPDS, 8.4% of the sample had a score of 13 and above, indicative of varying severity of depression (Cox et al., 1987). With respect to the PSQI, the average score, and percent above cutoff for significant sleep disturbances, was above previously published reports in samples of mothers of infants (e.g., Dørheim et al., 2009; Hairston et al., 2016), potentially due to the inclusion of mothers of infants only a few weeks old whose sleep patterns have not stabilized.

**Table 1:** Sample characteristics.

Demographic variables	Statistic
Education (%)	
• Up to 12 years	9.7
• Bachelor/attaining BA	52.4
• MA and higher	37.9
With partner/married (%)	98.4
Mother age (*M*[*SD*])	31.0 [3.5]
Infant age in months (*M[SD]*)	5.2 [3.3]
No. of children (*M[SD]*)	1.6 [0.9]
Primipara (%)	62.5
Pregnancy at risk (%)	16.7
Pregnancy complications (%)	19.2
Type of birth	
• Vaginal (%)	76.2
• Planned cesarean (%)	6.2
• Instrumental vaginal (%)	8.9
• Emergency cesarean (%)	7.9
Premature delivery (%)	4.2

**Self-report instruments**	**Statistic**

IES-R total sxs (*M[SD]*)	6.8 [9.9]
• IES intrusion (*M*[*SD*])	3.0 [4.3]
• IES avoidance (*M[SD]*)	1.9 [3.6]
• IES hyperarousal *(M[SD])*	2.1 [3.4]
EPDS (*M*[*SD*])	5.3 [4.5]
• Above 12 cutoff (%)	8.4
PSQI (*M*[*SD*]) (*N = 342*)	8.2 [3.0]
• Above 5 cutoff (%)	80.7

*Demographic data and descriptive analyses of instruments (*N* = 504). IES-R, Impact of Events Scale, Revised; EPDS, Edinburgh Postnatal Depression Scale; PSQI, Pittsburgh Sleep Quality Index. Note reduced number of respondents to PSQI*.

Due to a technical problem with responses to items referring to bedtime and wake-time, 162 participants’ responses were not recorded and hence excluded from the calculation of the summary PSQI score. To avoid excluding these participants in further analyses, the total PSQI score was correlated with each of the seven components of the scale, yielding the highest correlation [*R*_(340)_ = 0.696, *p* < 0.001] for component #6 (‘Subjective Sleep Quality’). Next Mann–Whitney *U* test was run to compare the distribution of responses on component #6 between participants for whom bedtime/wake-time responses were recorded vs. the subsample of missing responses. This analysis indicated that the distribution of the two groups, on component #6, did not differ (*p* = 0.767). Thus, further analyses were performed using component #6 of the PSQI.

**Table 2** summarizes the responses to the BiTS. Internal consistency of the ‘re-experiencing’ symptoms subscale was Cronbach α = 0.78, for the ‘avoidance’ symptoms subscale it was Cronbach α = 0.79, for ‘negative cognitions and mood’ subscale Cronbach α = 0.75, and for hyperarousal it was Cronbach α = 0.85. The proportion of women who meet DSM-V criteria for PTSD was 2.4% (*n* = 12), of these a third reported that their symptoms occurred during pregnancy (*n* = 4, data not shown). When including the negative emotions item, which comports with DSM-IV A2 criterion, these values remained constant (data not shown). Of women who endorsed having any symptoms of PTSD (approximately 54%), nearly a quarter reported experiencing symptoms during pregnancy, and 71% during the first 6 months after giving birth. Further, a plurality experienced PTSD symptoms for more than 3 months.

**Table 2:** Characteristics of responses to BiTS.

Item/subscale	Statistic
DSM-V criterion A (% positive)	12.9
DSM-IV criterion A2 (% positive)	12.3
Sum of Total symptoms scale (*M[SD]*)	8.5 [8.4]
• Mean Re-experiencing symptoms (*M*[*SD*])	0.30 [0.47]
• Mean Avoidance symptoms (*M*[*SD*])	0.21 [0.56]
• Mean Negative Mood and Cognitions (*M*[*SD*])	0.42 [0.47]
• Mean Hyperarousal symptoms (*M*[*SD*])	0.62 [0.64]
Dissociative symptoms (% positive)	8.5
Emotion numbing (% positive)	5.8
Total with symptoms (% positive)	54.6
• Before birth (%)	12.7
• First 6 months (%)	38.9
• More than 6 months after birth (%)	3.2
Duration of symptoms (% positive)	54.5
• <1 month (%)	12.3
• 1–3 months (%)	19.4
•≥3 months (%)	22.8
Symptom cause distress (% positive)	29.6
Symptoms impact normal functioning (% positive)	22.0
Symptoms due to other condition (% positive)	4.0
DSM-V criteria for PTSD (% positive)	2.4

*DSM-V Criterion A included two items; DSM-IV Criterion A2 was a single item. Total symptoms subscale included 23 items, ‘re-experiencing symptoms’ – 5 items, ‘avoidance symptoms – 2 items, ‘negative mood and cognitions’ – 7 items, ‘hyperarousal symptoms’ – 6 items. Two items addressed dissociative symptoms and one addressed emotional numbing, neither of which are necessary for diagnosis and were not used for determining DSM-V threshold for PTSD*.

### Exploratory Factor Analysis

The EFA resulted in a two-factor solution for the symptoms subscale of the BiTS (**Table 3**), which explained 46.0% of the total variance, and received an acceptable value on the Kaiser-Meyer-Olkin measure of sampling adequacy (KMO = 0.897, *p* < 0.001). Coefficients for 22 of 23 items exceeded 0.30, yielding two distinct factors, Factor 1 including items associated with general symptoms of distress, and Factor 2 including items assessing cognitions and emotions associated with the childbirth event. Two subscales were generated with internal consistency coefficients of Cronbach’s α = 0.90 for Factor 1, and Cronbach’s α = 0.85 for Factor 2.

**Table 3:** Factor solution of EFA.

Structure matrix	Factor 1	Factor 2
Q4. Recurrent unwanted memories of the birth…		-0.589
Q5. Bad dreams or nightmares about the birth…		-0.401
Q6. Flashbacks to the birth and/or reliving the experience		-0.399
Q7. Getting upset when reminded of the birth		-0.803
Q8. Feeling tense or anxious when reminded of the birth		-0.818
Q9. Trying to avoid thinking about the birth		-0.841
Q10. Trying to avoid things that remind me of the birth…		-0.682
Q11. Not able to remember details of the birth		
Q12. Blaming myself … for what happened during birth		-0.584
Q13. Feeling strong negative emotions about the birth …		-0.806
Q14. Feeling negative about myself …	0.666	
Q15. Lost interest in activities that were important to me	0.687	
Q16. Feeling detached from other people	0.743	
Q17. Not able to feel positive emotions …	0.712	
Q18. Feeling irritable or aggressive	0.754	
Q19. Feeling self-destructive or acting recklessly	0.666	
Q20. Feeling tense and on edge	0.786	
Q21. Feeling jumpy or easily startled	0.761	-0.303
Q22. Problems concentrating	0.697	
Q23. Not sleeping … not due to the baby’s sleep pattern	0.502	
Percent of variance	30.2	15.8
Cronbach’s alpha	0.90	0.85

### Effects of Background Variables

To assess the effects of demographic background on symptom severity, the total subscale of the BiTS and the two factors were correlated with maternal age, maternal education, and infant age. Of these variables only maternal education had a weak negative correlation with the total symptoms scale (*R* = -0.114, *p* = 0.010) and Factor 2 – birth-related symptoms (*R* = -0.138, *p* = 0.002), indicating that higher education was associated with less symptoms, specifically those regarding the birth itself.

**FIGURE 1:**
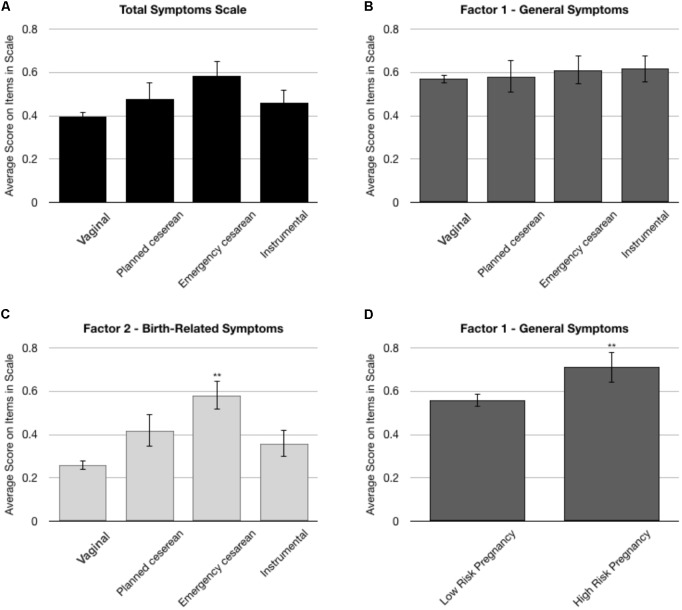
Univariate ANOVA analyses on the total symptoms subscale of the BiTS and the two Factors derived from the factor analysis. **(A–C)** Comparison of the effects of type of delivery on symptom severity. There was a main effect of delivery type on the total symptom subscale and on Factor 2, due to significantly higher scores among women who underwent emergency cesarean. **(D)** Effects of pregnancy risk on Factor 1. ^∗∗^*p* < 0.001.

Univariate ANOVAs were used to assess the effects of type of delivery (i.e., vaginal, planned cesarean, emergency cesarean, and instrumental) on symptom severity. There was a main effect of type of delivery on total symptoms [*F*_(3,496)_ = 2.80, *p* = 0.039, $\eta_{p}^{2}$ = 0.02, **Figure 1A**] and Factor 2 [*F*_(3,496)_ = 7.99, *p* < 0.001, $\eta_{p}^{2}$ = 0.05, **Figure 1C**], but not on Factor 1 [*F*_(3,496)_ < 1.0, **Figure 1B**]. This was due to higher scores among women who underwent emergency cesarean compared with other types of delivery (*post hoc* comparisons were all *p* < 0.001 after Bonferroni correction). Additionally, Univariate ANOVAs were used to assess the effects of high risk pregnancy, and giving birth prematurely. Women who reported a high risk pregnancy had higher score on Factor 1 [*F*_(1,503)_ = 4.33, *p* = 0.038, $\eta_{p}^{2}$ = 0.01, **Figure 1D**], but not on the total symptoms scale [*F*_(1,503)_ = 1.73, *p* = 0.189] or on Factor 2 [*F*_(1,503)_ < 1.0, data not shown]. Giving birth prematurely had no effect on any of the scales (data note shown).

### Construct Validity

To assess convergent validity, zero-order correlations were run with the BiTS subscales and factors and other dependent measures. Except for the correlations of PSQI C#6 (subjective sleep quality) with BiTS ‘re-experiencing’ and BiTS ‘avoidance,’ all correlations were significant, including after Bonferroni correction, and the majority were moderate to strong. As can be seen in **Table 4**, the ‘re-experiencing’ subscale had moderate and strong correlations with the BiTS ‘avoidance,’ BiTS Factor 2, and IES-R ‘avoidance’ and ‘intrusion’ symptoms; notably these latter two measures strongly correlated with each other. The BiTS ‘avoidance’ subscale had moderate correlations with the BiTS Factor 2 and IES-R ‘avoidance’ and ‘intrusion’ symptoms. The BiTS ‘negative mood and cognitions’ subscale had moderate correlations with the BiTS dissociative symptoms and with IES-R ‘avoidance’ and ‘hyperarousal symptoms, and strong correlations with Factor 1 and the EPDS. The BiTS ‘hyperarousal’ subscale had a moderate correlation with the BiTS dissociative symptoms and IES-R ‘hyperarousal’ symptoms, and strong correlations with Factor 1 and with EPDS. BiTS Factor 1 had moderate to strong correlations with IES-R ‘hyperarousal’ symptoms and EPDS, while Factor 2 had strong correlations with IES-R ‘avoidance’ and ‘intrusion’ symptoms. The correlations with PSQI component #6 were weak, however, as predicted the strongest correlation was with BiTS ‘hyperarousal.’

**Table 4:** Zero-order correlations.

		Re-experience	2	3	4	5	6	7	8	9	10	11	12
BiTS	2. Avoidance	0.617 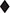	–										
	3. Mood	0.447 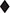	0.422 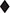	–									
	4. Hyperarousal	0.300 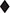	0.187^∗^	0.738 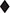	–								
	5. Dissociative	0.283 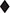	0.242 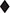	0.608 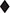	0.592 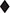	–							
	6. Numb	0.196^∗^	0.161^∗^	0.463 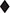	0.467 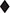	0.434 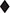	–						
	7. Factor 1	0.296 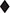	0.205 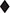	0.857 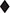	0.962 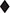	0.634 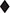	0.485 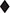	–					
	8. Factor 2	0.919 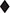	0.792 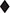	0.622 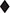	0.432 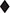	0.388 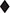	0.290 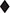	0.432 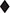	-				
IES-R	9. Avoidance	0.622 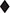	0.725 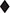	0.562 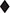	0.415 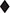	0.446 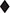	0.379 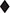	0.413 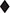	0.758 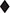	–			
	10. Intrusions	0.722 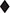	0.501 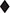	0.496 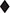	0.398 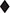	0.385 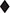	0.325 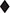	0.394 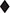	0.727 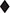	0.713 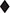	–		
	11. Hyperarousal	0.445 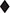	0.333 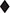	0.629 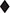	0.692 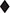	0.538 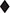	0.385 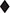	0.681 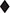	0.538 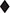	0.601 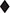	0.627 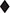	–	
	12. EPDS	0.347 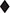	0.290 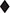	0.720 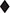	0.737 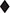	0.559 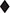	0.450 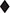	0.782 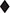	0.454 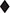	0.423 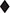	0.434 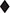	0.651 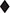	–
	13. PSQI C#6	0.08	0.02	0.259 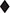	0.318 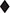	0.191^∗^	0.133^∗^	0.310 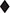	0.123^∗^	0.116^∗^	0.129^∗^	0.245 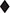	0.306 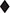

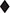
*Bonferroni correction *p* = 0.0007692; ^∗^*p* < 0.001*.

Fourteen variables and items were included in the TCSA procedure: From the BiTS the four symptom cluster subscales, the average of the items relating to dissociation, the emotional numbing item, the DSM-5 and DSM-IV criterion A items. Additionally, component #6 of the PSQI, the mean score on the EPDS, and the three IES-R subscales. The procedure yielded a two-cluster solution (BIC change = -1088.03; Ratio of distance measures = 1.00), with a fair cluster quality. Cluster 1 included 66.5% (*n* = 335) of the sample, and the other 33.5% (*n* = 169). **Table 5** lists the relative contribution of the different variables to the clustering solution. As can be seen, in cluster 1, the severity of symptoms on all scales was lower than the severity on cluster 2. Notably, more general symptoms (e.g., ‘negative mood and cognition’) were better predictors of cluster inclusion than birth related symptoms (e.g., ‘re-experiencing’).

**Table 5:** Two-step cluster analysis.

Subscale/item	Importance	Cluster 1 *M*[*SD*]/Mode[%]	Cluster 2 *M*[*SD*]/Mode[%]
BiTS negative mood and cognitions	1.00	0.23 [0.28]	0.80 [0.53]
EPDS	0.95	0.34 [0.89]	0.89 [0.50]
IES-R intrusions	0.77	1.38 [2.13]	6.07 [5.57]
BiTS ‘feeling emotionally numb’^∗^	0.75	“not at all” [99.7%]	“not at all” [57.4%]
BiTS hyperarousal	0.73	0.38 [0.41]	1.07 [0.77]
IES-R hyperarousal	0.71	0.85 [1.61]	4.44 [4.56]
BiTS ‘believe baby/self will be harmed’^∗^	0.65	“no” [100%]	“no” [65.1%]
IES-R avoidance	0.64	0.65 [1.33]	4.33 [5.19]
BiTS re-experiencing	0.60	0.14 [0.23]	0.60 [0.64]
BiTS avoidance	0.55	0.03 [0.18]	0.56 [0.83]
BiTS dissociative symptoms	0.45	0.15 [0.45]	0.93 [1.31]
BiTS ‘intense negative emotions’^∗^	0.44	“no” [69.6%]	“yes” [74.6%]
BiTS ‘fear of death’^∗^	0.26	“no” [100%]	“no” [85.8%]
PSQI component #6 (sleep quality)^∗^	0.09	“fairly good” [57.3%]	“fairly good” [45.0%]

*cluster 1, *n* = 335; cluster 2, *n* = 169. ‘^∗^’ single items*.

With respect to the remaining items concerning, onset, duration, severity and impact on functioning: 276 women (54.7%) endorsed having significant symptoms on these items, where the majority of cluster 2 (82.7%, *n* = 138) reported symptoms, compared with 41.2% (*n* = 138) of cluster 1 [χ^2^_(2)_ = 74.24, *p* < 0.001]. Chi square analyses on the distribution of the two clusters within the categories of onset and duration of symptoms indicated no difference between the two clusters [onset: χ^2^_(2)_ = 1.25, *p* = 0.54; duration: χ^2^_(2)_ = 3.68, *p* = 0.16, data not shown]. However, more women in cluster 2 endorsed significant distress [χ^2^_(2)_ = 23.67, *p* < 0.001], and nearly significant impact on functioning [χ^2^_(2)_ = 5.51, *p* = 0.060, data not shown].

## Discussion

The focus of this study was the validation of the Hebrew version of the City trauma scale (BiTS) in a community sample of women who gave birth in the past year. The BiTS is a new instrument designed to specifically evaluate childbirth related-PTSD symptoms according to DSM-5 criteria. The Hebrew version was found to be psychometrically sound with internal consistency for each of the symptom cluster subscales and total scale found, with Cronbach’s alpha values above acceptable level of 0.7. The present results also provide evidence of convergent validity for the BiTS, with its moderately strong relationship with IES-R subscales and EPDS scores, and clustering of participants with respect to the overall severity of their symptoms.

Using the BiTS, 2.4% of respondents met DSM-5 diagnostic criteria for PTSD, a percentage equivalent to those reported in recent systematic reviews and meta-analyses in community samples in other countries implementing validated PTSD instruments (Grekin and O’Hara, 2014; Dekel et al., 2017; Yildiz et al., 2017). Two recent studies reported prevalence in Israeli samples, both using the Post-Traumatic Stress Diagnostic Scale (PDS; Foa, 1995), a self-report questionnaire based on DSM-IV criteria. In one study, a sample of 89 women surveyed 1 month postpartum, found the prevalence of women meeting full diagnostic criteria to be 3.4%, 7.9% having sub-clinical PTSD symptoms, and 25.9% significant symptoms of PTSD (Polachek et al., 2012). A subsequent study by the same group, in a sample of women with high-risk pregnancies, found the prevalence of full postpartum PTSD to be 9.9% and sub-clinical PTSD – 11.9%, 1-month postpartum (Polachek et al., 2016), suggesting that high risk pregnancies may increase the risk for developing childbirth-related PTSD. The slightly lower percentage of women meeting DSM-5 criteria in this study compared with the first of the two Israeli studies may be due to several factors, including: the use of a different instrument, the longer average delay between childbirth and data collection, and/or the fact that the present sample was relatively highly educated, which is considered protective against the development of PTSD (Brewin et al., 2000).

It should be further noted that 55% of the sample endorsed some symptoms of PTSD, of whom 30% endorsed significant distress, and 22% endorsed a negative impact on normal functioning. These numbers match those reported in other studies. For example, Davies et al. (2008) assessed 211 women 6 weeks postpartum for symptoms of PTSD, finding rates of full PPTSD at 3.8% and partial at 21.3%. In their meta-analysis, Dekel et al. (2017) found that the highest reported rates of partial PPTSD to be 27.3% in community sample.

### Exploratory Factor Analysis

Although the BiTS symptoms scale consists of the four DSM-5 symptoms cluster – re-experiencing, avoidance, negative cognitions and mood, and hyperarousal – we opted for an EFA due to the absence of previous studies investigating the factor structure of the instrument. The results of the EFA indicated a two-factor solution of the symptoms items, with one factor including items associated with the event of childbirth (Factor 2) and the other associated with more general symptoms (Factor 1), likely reflecting the phrasing of the questions. Notably the integrity of the subscales pertaining to three of the symptom clusters was retained, with ‘avoidance’ and ‘re-experiencing’ in one factor, and ‘hyperarousal’ symptoms in the other. In contrast, the new DSM-5 ‘negative mood and cognition’ cluster was split across the two factors.

This two-factor solution corresponds with the structure of the original scale (Ayers et al., 2018) and with two previous studies using different scales to assess childbirth related PTSD (Ayers et al., 2009; Stramrood et al., 2010). In one study, Stramrood et al. (2010) found a two-factor solution using both the TES (Wijma et al., 1997) and the PTSD symptom scale short version (PSS-SR; Foa et al., 1993), a generic PTSD measure adapted according the stressor of interest. As in the present study, one of the factors was comprised of childbirth-related items, mainly intrusions and avoidance, and the other factor compromised from general items mainly regarding hyperarousal and numbing. In the second study, the general PDS (Foa et al., 1997) was used, but participants answered it regarding childbirth. In this study, two factors were also found, one symptom cluster for arousal and numbing and the other for re-experiencing and avoidance (Ayers et al., 2009). Although this two factor solution is not commonly found in studies of other traumas, it was also obtained in studies of United Nations peacekeepers and victims of motor vehicle accidents (Buckley et al., 1998; Taylor et al., 1998; Asmundson et al., 2003).

The difference between the more general and childbirth-related symptoms is further demonstrated by the fact that the mode of delivery (emergency CS vs. other modes of delivery) was associated with Factor 2 (childbirth-related symptoms) whereas Factor 1 (general symptoms) was associated with a high-risk pregnancy. Several reviews and meta-analyses have implicated emergency cesarean and high risk pregnancies as risk factors for childbirth-related PTSD (Andersen et al., 2012; Ayers et al., 2016; Dekel et al., 2017). Our findings add to this observation, suggesting that the trajectory and consequences of stress during pregnancy vs. stress during delivery maybe different, with the former having greater impact on more general dysphoria though further research is needed.

### Construct Validity

The correlation analysis confirmed that the symptom clusters delineated by the BiTS correspond to symptoms measured using other instruments. Thus, the subscales of the ‘hyperarousal,’ ‘avoidance,’ and ‘re-experiencing’ clusters were highly correlated with the equivalent subscales on the IES-R (hyperarousal, avoidance, and intrusions, respectively). Similarly, ‘negative mood and cognitions’ strongly correlated with the EPDS, while the highest correlation with subjective sleep difficulties was with the BiTS ‘hyperarousal’ cluster. The two factors detected in the EFA correlated differentially with the IES-R, EPDS, and PSQI, wherein Factor 1 correlated more strongly with EPDS and PSQI, consistent with the notion that it reflected general dysphoria and hyperarousal, while Factor 2 had stronger correlations with the IES-R, specifically the avoidance and intrusions subscales, consistent with the observation that this factor was associated more strongly with birth-related cognitions.

Finally, of late, focus has shifted away from specific clinical diagnoses toward the identification of psychological constructs that underlie psychopathological phenomena, which may more closely ally with biological and developmental processes. It has been the emphasis in clinical and preclinical research (Sanislow et al., 2010). Our cluster analysis differentiated two groups within the sample that differed across all measures collected. The majority of cluster two (82.7%) reported having significant symptoms, compared with 41.2% of cluster 1, and more women in cluster 2 endorsed significant distress and impact on functioning. Clustering was more strongly influence by measures of dysphoria and hyperarousal, while more specific symptoms contributed less to the clustering solution. This relationship is consonant with the two factors of the BiTS, despite not being included in the cluster analysis, suggesting that symptoms relating to specific aspects of the trauma differ qualitatively from general symptoms in their contribution to the manifestation of the pathology. Arguably, in a clinical sample, with a greater representation of women with clinically significant symptoms, the outcome of the analysis may have favored specific rather than general symptoms.

It should be noted that the IES-R intrusions subscale also contributed to the differentiation of the clusters, although this measure correlated more strongly with childbirth-related symptoms. Potentially, this was due to items pertaining to emotions in this subscale (e.g., “I had waves of strong feelings about it”). As noted above, due to the inability to avoid a key reminder of the childbirth event (namely, the child), it would be expected that intrusions would be a central feature of childbirth-related PTSD, relative to other forms of PTSD. In addition, avoidance-related negative emotions tend to generalize to trauma-unrelated environmental cues, which in turn may trigger intrusions (Schmidt and Vermetten, 2017).

Finally, it should further be noted that emotional numbing was the fourth significant variable distinguishing the clusters. This symptom was recently omitted from DSM-5 criteria despite evidence to suggest that emotional numbing is strongly predictive of the severity of PTSD (Hoge et al., 2016), and may be more predictive of parenting stress than other PTSD symptoms (Wilson et al., 2017). In sum, the cluster analysis suggests that at least in non-clinical samples, childbirth-related PTSD may manifest as more general dysphoric symptoms and hyperarousal rather than re-experiencing and avoidance, underscoring the importance of measuring these aspects of PTSD in postpartum mothers.

### Limitations

Some limitations to this study should be addressed in terms of the study’s procedure and participants. First, although web-based questionnaires may promote more authentic disclosure of undesirable behaviors or attitudes, as an online setting enhances perceived anonymity (Crutzen and Göritz, 2010), such methodology also limits the ability to verify the accuracy of data. Second, the sample may not be representative as the nature of access to online services, and the willingness and time availability to invest in completing a long survey may result in selective study participation. Third, as this was an exploratory study, we did not measure test–retest reliability. Finally, the sample was non-clinical, while the BiTS is aimed at detecting clinically significant PTSD. Future studies are necessary, in high risk populations, and populations diagnosed with PPTSD using the gold standard of clinical interview.

## Conclusion

As our understanding of PPTSD is still emerging, the awareness that this disorder affects the lives of many women is increasing. Further, as childbirth as a potentially traumatic event may be distinct from other traumas, and related post-trauma symptoms may differ from other PTSD phenomenology, there is a need for a designated scale to measure PPTSD, which both meets clinically guidelines but which hones also in to the unique characteristics of PPTSD. The City BiTS Hebrew version is potentially a useful tool in both research and clinical arenas. It demonstrated high internal consistency for the total scale and for the symptom cluster subscales. An EFA yielded a two-factors solution, accounting for 45% of variance, with two factors of general symptoms and childbirth-related symptoms both demonstrating high internal consistency. Further, High convergent validity for the symptom cluster subscales was demonstrated with the parallel IES-R subscales, EPDS and PSQI. Finally, a TSCA indicated that dysphoric and hyperarousal symptoms best differentiated the severity of symptoms of respondents across measures. Further research is needed in high-risk populations, as well as validation studies against PTSD clinical interviews are warranted.

## Ethics Statement

This study received approval from the Academic College of Tel Aviv-Yaffo Research Ethics Committee. Reference: 201617. Date of approval: 7.12.2016. All subjects gave written informed consent in accordance with the Declaration of Helsinki.

## Author Contributions

JH planned the project, was in charge of the study design and jointly headed the manuscript writing. AM contributed to the study design and collected the data. IH did the statistical analyses and jointly headed the manuscript writing. JH and IH contributed equally to the writing of the manuscript.

## Conflict of Interest Statement

The authors declare that the research was conducted in the absence of any commercial or financial relationships that could be construed as a potential conflict of interest.

## Footnotes

1 http://www.qualtrics.com
